# Supplementary material for: α-Synuclein Induces Neuroinflammation Injury through the IL6ST-AS/STAT3/HIF-1α Axis
Source: Int J Mol Sci. 2023 Jan 11;24(2):1436. doi: 10.3390/ijms24021436 (PMC9861378; doi:10.3390/ijms24021436)
Supplement: Supplementary file 1 [file ijms-24-01436-s001.zip › ijms-2048136-supplementary.pdf]

Supplement data

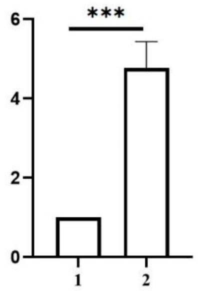

Figure S1. The expression of IL6 was increased in α-synuclein-induced HM cells

Detect the expression of IL6 by qPCR in control HM cells and α-synuclein-induced HM cells. Its quantitative data is displayed as mean ± SD, *** indicates $P < 0.001$. 1. the control group, 2. α-synuclein induced group.

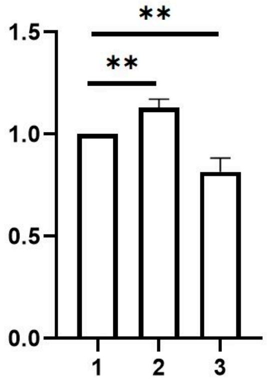

Figure S2. The influence upon oxidate stress in α-synuclein-treated SH-SY5Y cells

Detect the ROS content by flow cytometry in control SH-SY5Y cells and α-synuclein-induced SH-SY5Y cells. Its quantitative data is displayed as mean ± SD, ** indicates $P < 0.01$. 1. the control group, 2. α-synuclein induced the cells for 1h, 3. α-synuclein induced the cells for 24h.

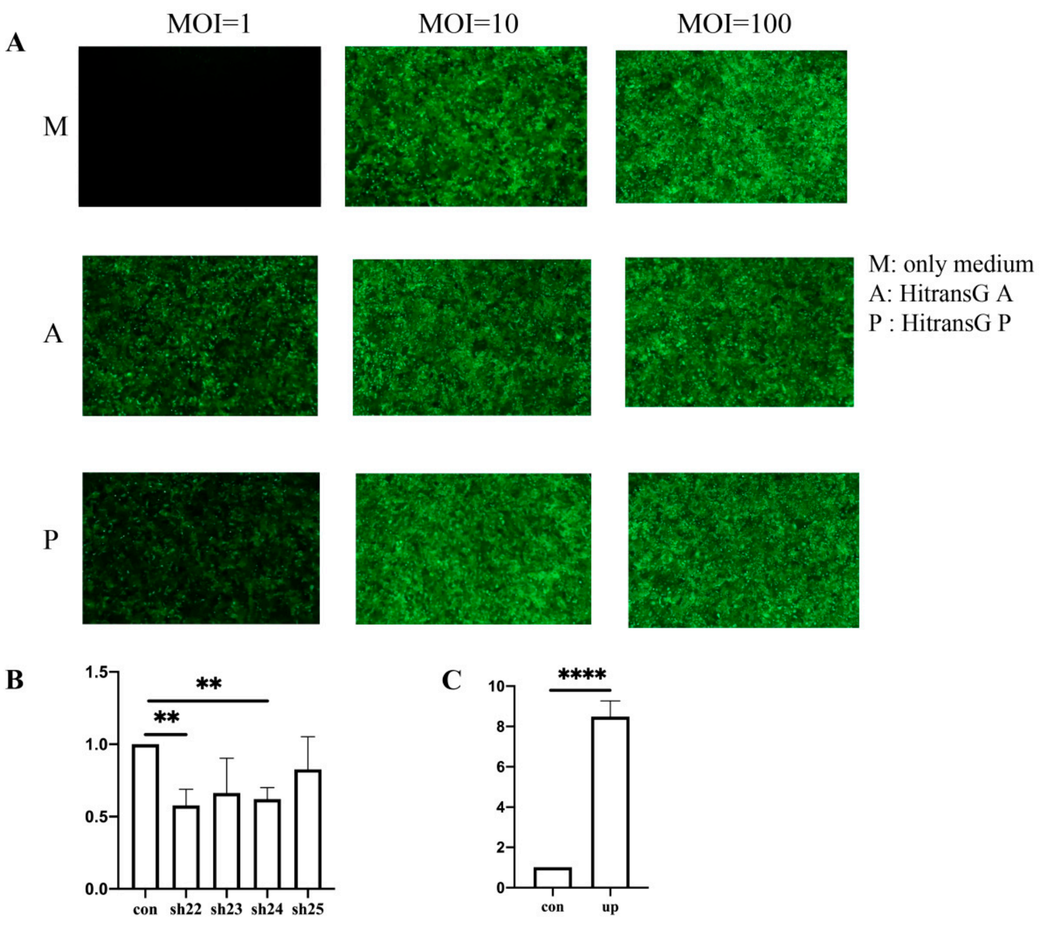

Figure S3. RNA Transfection and Interference process

(A) Suitable transfection reagent and multiplicities of infection (MOI) was screened. (B) Most efficient shRNA sequence towards IL6ST-AS among 4 shRNA constructs (sh22, sh23, sh24 and sh25) was screened by quantitative real-time PCR (qRT-PCR). (C）Efficiency of overexpression plasmid towards IL6ST-AS (IL6ST-AS up) was validated by qRT-PCR.

Table S1. The demographic description of PD patients 1

The PD patients 1 (Huang) have been brought into the clinical follow-up from 2015. Her clinical characteristic was analysis and recorded in 2015, 2016, 2017 and 2018.

		Huang Ruxiang Female 65Y(2018)			
		First Time 2015.03.03	follow-up I 2016.07.05	follow-up II 2017.06	follow-up III 2018.09
Course of disease		1 year	2 years	3 years	4 years
Main symptoms	Bradykinesia	Bradykinesia	Bradykinesia	Bradykinesia	Bradykinesia
	Myotonia	The muscular tension of both upper limbs is increased, and that of	The muscular tension of both upper limbs is increased, and that of	The muscular tension of both upper limbs is increased,	The muscular tension of both upper limbs is increased, and that of
	tremor	/	Both hands static tremor	Both hands static tremor	Both hands static tremor
	Postural disorder	/	/	/	It is easy to fall when starting or turning, and the arm swing while walking is reduced
Other motor symptoms	gait disturbance	Flustered gait	Flustered gait	Unstable walking and flustered gait	Unstable walking and flustered gait
	Masked face	less	less	less	less
	Micrographia	/	/	/	/
	Difficulty in turning over	/	/	/	/
	Fall or unsteadiness	/	/	/	easy to fall down
	Linguistic difficulties	/	/	/	/
	Dysphagia	/	/	/	/
Autonomic symptoms	Hydrostomia	/	/	/	/
	sweat	/	/	/	/
	Seborrhagia	/	/	/	/
	Constipation	Yes, need to be helped by catharsis drugs	Yes, need to be helped by catharsis drugs	Yes, need to be helped by catharsis drugs	Yes, need to be helped by catharsis drugs
	Frequent urination, urgency of urination, urinary incontinence	/	/	/	/
	Orthostatic hypotension	/	/	/	/
	Somnipathy	Difficult to fall asleep, dreamy, easy to wake up	Difficult to fall asleep, dreamy, easy to wake up	/	Difficult to fall asleep, dreamy, easy to wake up
	Sexual dysfunction	/	/	/	/
Other symptoms	Cognitive Modifiability	/	/	/	Slow to react, decreased computing ability and independent living
	Personality changes	/	/	/	
	Dysosmia	/		/	Yes
	Depression	Dizziness/chest tightness/palpitation	Dizziness/chest tightness/palpitation	Dizziness/chest tightness/palpitation	/
	Anxiety	/	Anxiety and nervousness	Anxiety and nervousness	Anxiety and nervousness
Atypical symptoms	Cerebellar dysfunction	/	/	/	/
	Pyramidal sign	positive	positive	positive	positive
	Nystagmus	/	/	/	/
	Blepharospasm	/	/	/	/
	Eyelid disuse	/	/	/	/
	Hypermotor seizure	/	/	/	/
	Hallucination	/	/	Visual hallucination	Visual hallucination
Motor Complications	Dyskinesia	/	/	The hands and feet twist involuntarily	The hands and feet twist involuntarily
	End of dose phenomenon	/	/	/	
	On-off phenomenon	/	/	/	
	Morning stiffness	/	/	/	
	Painful spasm	/	With involuntary muscle pain	With involuntary muscle pain	With involuntary muscle pain
	Other style of dyskinesia	/	/	/	/
Scale evaluation	UPDRS	23	34	54	77
	UPDRS-III	11	15	20	31
	Revised Hoehr l and Yahr stages	2	2	2.5	3
	MMSE	26	24	24	22
	MoCA	21	20	18	15
	Hamilton Depression Scale(HAMD)	12	14	14	8
	Hamilton Anxiety Scale(HAMA)	6	11	12	18
	Pittsburgh sleep quality index (PSQI)	9	9	6	13

Table S2. The demographic description of PD patients 2

The PD patients 2 (Li) have been brought into the clinical follow-up from 2015. His clinical characteristic was analysis and recorded in 2015 and 2018.

		Li Hanzhi Male 80Y(2018)	
		First Time	follow-up I
		2015.04.13	2018.10.31
Course of disease		3 years	6 years
Main symptoms	Bradykinesia	fingers were stiffening up and slowness of movements in both legs	fingers were stiffening up and severe slowness of movements in both legs
	Myotonia	High muscular tension of both upper limbs	High muscular tension of both upper limbs
	tremor	/	/
	Postural disorder	leaning forward	leaning forward
Other motor symptoms	gait disturbance	Flustered gait	Flustered gait
	Masked face		
	Micrographia	Yes	Yes
	Difficulty in turning over		
	Fall or unsteadiness		
	Linguistic difficulties		
	Dysphagia		Yes
Scale evaluation	UPDRS	22	36
	UPDRS-III	15	21
	Revised Hoehr 1 and Yahr stages	2.5	2.5
	MMSE	18	14
	MoCA	17	15
	Hamilton Depression Scale(HAMD)	8	14
	Hamilton Anxiety Scale(HAMA)	14	13
	Pittsburgh sleep quality index (PSQI)	5	7

Table S3. The demographic description of PD patients 3

The PD patients 3 (Li) have been brought into the clinical follow-up from 2019. Her clinical characteristic was analysis and recorded in 2019.

		Li Qunzhu Female 65Y
		follow-up I
		2019.11.21
Course of disease		14 years
Main symptoms	Bradykinesia	Slowness of movements
	Myotonia	High muscular tension of limbs
	tremor	upper limbs
	Postural disorder	leaning forward
Other motor sym ptoms	gait disturbance	Flustered gait
	Masked face	
	Micrographia	Yes
	Difficulty in turning over	Yes
	Fall or unsteadiness	Yes
	Linguistic difficulties	
	Dysphagia	
Autonomic sympt oms	Hydrostomia	
	sweat	
	Seborrhagia	
	Constipation	
	Frequent urination, urgency of urination, urinary incontinenc	Yes
	Orthostatic hypotension	
	Somnipathy	
	Sexual dysfunction	
Other symptoms	Cognitive Modifiability	
	Personality changes	
	Dysosmia	Yes
	Depression	
	Anxiety	
Scale evaluation	UPDRS	80
	UPDRS-III	42
	Revised Hoehr 1 and Yahr sta ges	4
	MMSE	20
	MoCA	16
	Hamilton Depression Scale(HAMD)	11
	Hamilton Anxiety Scale(HAMA)	16
	Pittsburgh sleep quality index (PSQI)	9

Table S4. The demographic description of PD patients 4

The PD patients 4 (Liu) have been brought into the clinical follow-up from 2019. Her clinical characteristic was analysis and recorded in 2019.

		Liu Yongtian Male 53Y<br>follow-up I<br>2019.12.10
Course of disease		4 years
Main symptoms	Bradykinesia	slowness of movements
	Myotonia	High muscular tension of upper limbs
	tremor	resting tremors in left hand
	Postural disorder	
Other motor sym ptoms	gait disturbance	
	Masked face	
	Micrographia	Yes
	Difficulty in turning over	
	Fall or unsteadiness	
	Linguistic difficulties	Yes
	Dysphagia	
Autonomic sympt oms	Hydrostomia	
	sweat	
	Seborrhagia	
	Constipation	Yes
	Frequent urination, urgenc y of urination, urinary inc ontinence	
	Orthostatic hypotension	
	Somnipathy	
	Sexual dysfunction	
Other symptoms	Cognitive Modifiability	Yes
	Personality changes	Yes
	Dysosmia	
	Depression	Yes
	Anxiety	Yes
Scale evaluation	UPDRS	74
	UPDRS-III	36
	Revised Hoehr 1 and Yahr stages	3
	MMSE	23
	MoCA	17
	Hamilton Depression Scale(HAMD)	14
	Hamilton Anxiety Scale(HAMA)	15
	Pittsburgh sleep quality index (PSQI)	14

Table S5. The detail information of normal control group

Age	Sex	diagnosis	biochemical test result of CSF
33	female	Tension headache	negative
56	female	vegetative nerve functional disturbance	negative
50	female	headache	negative
39	male	Bilateral middle cerebral artery stenosis	negative
